# Trion fine structure and coupled spin–valley dynamics in monolayer tungsten disulfide

**DOI:** 10.1038/ncomms12715

**Published:** 2016-09-02

**Authors:** Gerd Plechinger, Philipp Nagler, Ashish Arora, Robert Schmidt, Alexey Chernikov, Andrés Granados del Águila, Peter C.M. Christianen, Rudolf Bratschitsch, Christian Schüller, Tobias Korn

**Affiliations:** 1 Institut für Experimentelle und Angewandte Physik, Universität Regensburg, D-93040 Regensburg, Germany; 2 Physikalisches Institut, Westfälische Wilhelms-Universität Münster, D-48149 Münster, Germany; 3 High Field Magnet Laboratory (HFML—EMFL), Radboud University, 6525 ED Nijmegen, The Netherlands

## Abstract

Monolayer transition-metal dichalcogenides have recently emerged as possible candidates for valleytronic applications, as the spin and valley pseudospin are directly coupled and stabilized by a large spin splitting. The optical properties of these two-dimensional crystals are dominated by tightly bound electron–hole pairs (excitons) and more complex quasiparticles such as charged excitons (trions). Here we investigate monolayer WS_2_ samples via photoluminescence and time-resolved Kerr rotation. In photoluminescence and in energy-dependent Kerr rotation measurements, we are able to resolve two different trion states, which we interpret as intravalley and intervalley trions. Using time-resolved Kerr rotation, we observe a rapid initial valley polarization decay for the A exciton and the trion states. Subsequently, we observe a crossover towards exciton–exciton interaction-related dynamics, consistent with the formation and decay of optically dark A excitons. By contrast, resonant excitation of the B exciton transition leads to a very slow decay of the Kerr signal.

## Abstract (web summary)

Monolayer transition metal dichalcogenides are promising materials for valleytronics applications. Here, the authors study WS_2_ samples using photoluminescence spectroscopy and time-resolved Kerr-rotation measurements at low temperatures, gaining insight into the valley dynamics of excitons.

## Graphical abstract

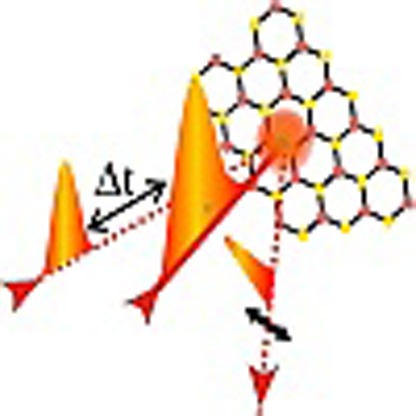

In recent years, two-dimensional crystal structures have emerged as a fascinating new field of materials science. Owing to the easy fabrication via simple mechanical exfoliation, a variety of different material classes is readily available as a two-dimensional sheet^1^, including large-gap insulators, superconductors and semiconductors, and the electronic structure of these ultimately thin layers can be very different from that of their corresponding bulk crystals. MoS_2_ and related transition-metal dichalcogenides (TMDCs) such as WS_2_ are among the most promising systems, while they are indirect-gap semiconductors in the bulk, a transition to a direct bandgap situated at the K points in the Brillouin zone occurs in single layers^2^,^3^,^4^. Owing to the spatial and dielectric confinement, the optical spectra are governed by excitonic features, which are stable even at room temperature and exhibit anomalously large binding energies of about 0.5 eV (refs 5, 6, 7, 8). This strong confinement also allows for the formation of more complex quasiparticles^9^, such as trions^10^,^11^,^12^, consisting of, for example, two electrons and a hole, and biexcitons^13^,^14^,^15^,^16^, consisting of two electrons and two holes, with binding energies ranging from about 30 to 70 meV. The peculiar band structure with its non-equivalent valleys has direct consequences for the trions, which may form optically bright states using carriers located within one valley (intravalley trions) or in different valleys (intervalley trions). An energetic splitting (trion fine structure) between these states due to exchange interaction was recently predicted and observed for WSe_2_ (refs 17, 18).

The spin and the valley pseudospin indices are coupled due to inversion symmetry breaking. Together with spin–orbit coupling, this results in an out-of-plane valley-contrasting spin splitting in, both, valence and conduction bands^19^,^20^. While the valence-band spin splitting ranges from about 150 (MoS_2_) to 450 meV (WSe_2_) in the TMDC monolayers, the conduction-band spin splitting is significantly smaller and shows a larger relative variance between the different TMDCs. For WS_2_ and WSe_2_, it has been theoretically predicted to be very large (−32 and −37 meV, respectively, an order of magnitude larger than for MoS_2_)^21^. The negative sign of the splitting indicates that the allowed interband transition from the upper valence band addresses the higher-energy conduction-band state in the tungsten-based TMDCs^22^. Recently, experimental evidence for the different sign of the conduction-band spin splitting in molybdenum- and tungsten-based TMDCs was found in temperature- and time-resolved photoluminescence (PL) measurements by several groups^23^,^24^,^25^,^26^. The optical selection rules for interband transitions enable valley-selective excitation of electron–hole pairs via circularly polarized light^19^,^20^, and the optically oriented valley polarization can be directly read-out using helicity-resolved PL^27^,^28^,^29^.

Monolayer TMDCs thus have emerged as promising candidates for valleytronic applications, where the spin degree of freedom is directly coupled to a specific valley and stabilized via large spin splittings. Therefore, detailed knowledge about the spin dephasing mechanisms and lifetimes is required for the realization of future devices. The theory of spin and valley relaxation mechanisms in TMDCs is currently under intense investigation^30^,^31^,^32^,^33^,^34^,^35^. The experimental study of spin and valley polarization dynamics via time-resolved PL measurements has turned out to be challenging because of an ultrafast radiative decay of the excitons^36^,^37^. Time- and helicity-resolved pump–probe measurements in MoS_2_ and WS_2_ monolayers grown by chemical vapour deposition have revealed exciton valley lifetimes of only few picoseconds at liquid nitrogen temperatures^30^,^38^,^39^. Recently, it was also demonstrated in a helicity-resolved pump–probe experiment on WS_2_ that Coulomb-induced intervalley coupling leads to an immediate and prominent optical response of the unpumped valley^40^. Time-resolved Kerr rotation (TRKR) measurements on mechanically exfoliated MoS_2_ at 77 K (ref. 41) and WSe_2_ at liquid helium temperatures^42^,^43^ yield very short exciton valley lifetimes on the picosecond timescale. Very recently, TRKR measurements on heavily doped chemical vapour deposition-grown disulfides and diselenides have shown spin lifetimes on the order of nanoseconds for resident electrons^44^,^45^ and holes^46^.

Here we combine low-temperature PL measurements with TRKR to study excitonic transitions in mechanically exfoliated single-layer WS_2_. We are able to resolve an energetic splitting of the trion resonance. We attribute this trion fine structure to the splitting between optically bright singlet and triplet trions, which arises due to (predominantly electron–hole) exchange interaction. Furthermore, we investigate the coupled spin–valley dynamics of A excitons, trions and B excitons. We observe that A excitons and trions show a rapid decay of the valley polarization on the few-picoseconds scale, and identify a crossover towards exciton–exciton interaction-related decay dynamics due to the formation of a subset of optically dark A excitons. By contrast, resonant excitation of the B exciton transition leads to a very slow decay of the Kerr signal.

## Results

### Observation of trion fine structure and valley polarization

**Figure 1:**
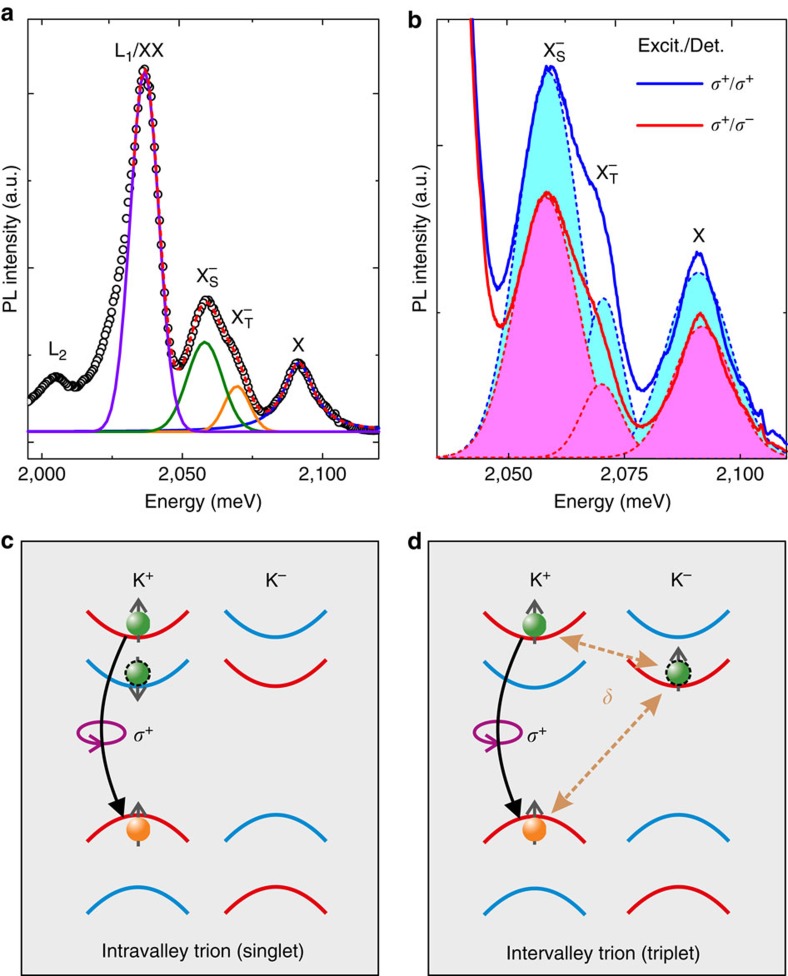
Trion fine structure and valley polarization observed in low-temperature PL. (**a**) PL spectrum of monolayer WS_2_ at a temperature of 4 K. The black circles represent the data, the red curve is the sum of the fit functions for neutral A excitons X (blue), the peaks we associate with triplet 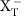 (orange) and singlet trions 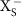 (green), and the biexciton/localized exciton complex XX/L_1_ (violet). In addition, a second localized exciton peak L_2_ at lower energies is visible. (**b**) Helicity-resolved PL spectra of the same flake as depicted in (**a**). The solid blue (red) lines represent the data for co-(contra-)circular excitation (Excit.) and detection (Det.), the dashed lines in corresponding colours are fit functions for X, 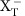 and 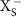. Circular polarization degrees of the different excitonic features are calculated by evaluating the areas under the corresponding fit curves. (**c**,**d**) Schematic illustration of singlet (**c**) and triplet (**d**) trion configurations for WS_2_ in the K^+^ valley. Electrons in the conduction (valence) band are represented by green (orange) spheres. Spin-up (-down) bands appear in red (blue) colour. The curved arrows indicate the interband recombination. The final state of the remaining conduction-band electron after trion recombination is marked by the dashed black outline. The intervalley electron–hole exchange interaction leading to an energetic splitting *δ* is indicated by the dashed arrows.

First, we discuss the PL measurements. Figure 1a shows a PL spectrum of a monolayer WS_2_ flake measured at liquid helium temperature. The spectrum is comprised of several peaks. The highest-energy peak at about 2,091 meV corresponds to the neutral A exciton transition (labelled X). The feature around 2,065 meV can be identified as the negatively charged exciton (labelled X^−^), its fine structure will be discussed below. The energetic splitting between the neutral and charged exciton allows us to determine the background carrier concentration in our sample (Supplementary Note 1). The large peak at 2,036 meV consists of a superposition of biexciton emission (labelled XX), which is observable in WS_2_ even under continuous-wave illumination, and defect-bound exciton emission (labelled L_1_). Another defect-bound exciton emission peak (labelled L_2_) is observable at lower energy. The energetic positions of exciton, trion and biexciton are in good agreement with previous studies on monolayer WS_2_, where their assignment, including the negative charge state of the trion, was confirmed in gate-voltage- and excitation-density-dependent PL measurements^15^. Remarkably, in this spectrum, the trion feature clearly shows a substructure. We note that this substructure is clearly observable in PL spectra recorded at different positions of our sample (Supplementary Fig. 1). In contrast to the XX and X features, the trion peak cannot be described by a single peak function, but only by a superposition of two Gaussians, which are shifted in energy by 11 meV. The sum of these four peak functions (dashed red line) closely matches the experimental data set (open circles). We attribute the two Gaussians needed to describe the trion feature to the two optically bright, negatively charged trion species that may form in monolayer WS_2_. These states are sketched in Fig. 1c and d, respectively: intravalley trions, in which a hole and two electrons in the same valley form a bound trion state, and intervalley trions, where the two constituent electrons are located in different valleys. For intravalley trions, the Pauli exclusion principle dictates that the two electrons must occupy different spin states and therefore reside in the top and bottom spin-split conduction bands, respectively, forming a spin singlet. For intervalley trions, by contrast, the two electrons may have the same spin, as they differ in their valley index, forming a spin triplet. For both optically bright trions, the optical selections rules enforce a recombination of the electron in the higher-energy spin-split conduction band with the hole in the same valley, so that an electron in the lower spin-split conduction band remains. To explain the energetic difference between the two trion species, we need to consider the exchange interaction of the excess electron with the electron–hole pair in the opposite valley in intervalley trions, in which all three constituent particles have the same spin orientation. This effect was first discussed for monolayer WSe_2_ by Yu *et al.*^17^. Owing to Coulomb repulsion, the electron–electron exchange in the intervalley trion is much smaller than the exchange interaction of electron and hole. Overall, the exchange interaction leads to an increase of the triplet state energy by the value *δ*, which was estimated to be on the order of 6 meV (ref. 17) for WSe_2_. The overall effect is a splitting of the trion dispersion into two branches, the upper one for triplet and the lower one for singlet states, separated by the energy difference *δ*. Recently, an energy splitting of the trion PL emission in good agreement with this calculation was reported for monolayer WSe_2_ (ref. 18).

Further evidence for the existence of the two different trion species is observable in helicity-resolved PL spectra: Fig. 1b shows PL spectra of the same flake as in Fig. 1a, for co- (solid blue line) and contra-circular (solid red line) excitation and detection, respectively. Here we focus only on the trion and neutral exciton features. For both spectra, we utilize two Gaussians to fit the trion feature, and a single Lorentzian for the neutral exciton, with the sum of the three peak functions (not shown) closely matching the experimental data. Both, exciton and trion PL emission are more intense for co-circular excitation and detection, indicating a pronounced optically induced valley polarization. By directly comparing the spectra, one can observe that the higher-energy triplet trion emission is more strongly quenched for contra-circular excitation and detection than the lower-energy singlet trion. To quantify this observation, we determine the PL emission intensity of all three peaks by calculating the integrated intensity of each peak function (indicated by the shaded areas under the curves). From these values, we are able to determine the circular polarization degree of the different species, *P*_Circ_=(*I*_Co_−*I*_Contra_)/(*I*_Co_+*I*_Contra_). Remarkably, we find that for the two trion species, the circular polarization degree varies by a factor of almost two: while the peak we associate with the singlet trion shows a value of about 19%, similar to the neutral exciton (21%), the peak we attribute to the triplet trion shows a significantly larger value of 34%, strengthening our assignment of the two emission features to different trion species. Again, this observation is in qualitative agreement with the recent report of trion fine structure in WSe_2_ (ref. 18), where it was related to the different dispersions of the two trion species leading to a smaller spread in momentum space for the higher-energy triplet trion. We note that in continuous-wave PL experiments, the time-integrated circular polarization degree is detected. This value depends on a combination of valley dephasing and photocarrier recombination rates, both of which may be different for the different trion species. We will discuss these dynamics in more detail below.

### Study of excitonic resonances in Kerr rotation experiments

**Figure 2:**
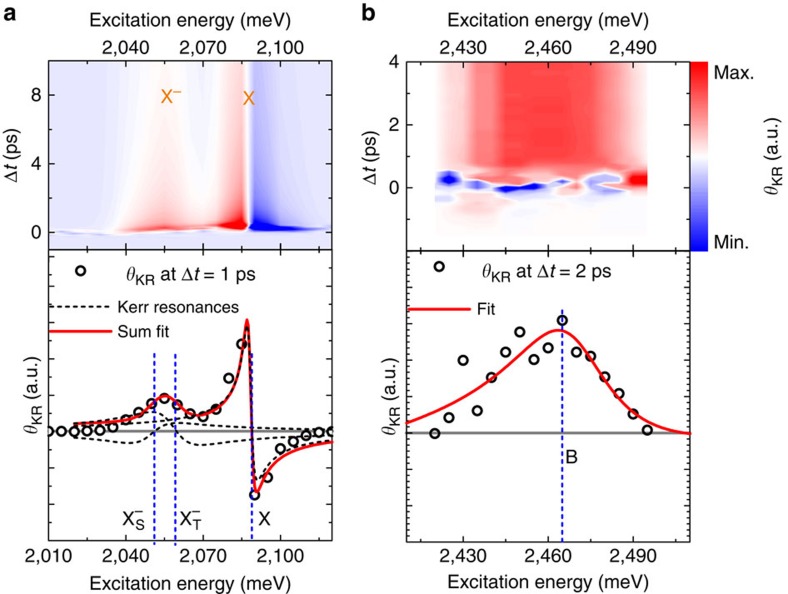
Resonant excitation of valley-polarized exciton and trion states. Time- and excitation-energy-resolved Kerr rotation measurements data in the energy range of (**a**) the A exciton resonance and (**b**) the B exciton resonance. The lower panels depict the Kerr rotation angle at a fixed time delay after pulsed excitation. Here the solid red lines indicate the fits to the data, while the blue dashed lines mark the energetic positions of trions and excitons. In the lower left panel, the black dashed lines indicate the contributions of the three resonances considered in the fit.

We now turn to our TRKR spectroscopy experiments. First, we discuss the laser energy dependence of the Kerr signals. The energy dependence of the Kerr rotation signal has been successfully used in recent years to study free carrier, exciton and trion spin dynamics in various semiconductor heterostructures^47^,^48^,^49^,^50^. Figure 2 shows two series of TRKR measurements, in which our pulsed laser system was tuned in the energy ranges of the A exciton (Fig. 2a) and the B exciton (Fig. 2b). The upper panels depict the Kerr rotation angle *θ*_KR_ as a function of time delay between the pump and probe pulses and the laser energy in a false colour plot, while the lower panels present cuts for fixed time delays shortly after the arrival of the pump pulse. The energy dependence of the Kerr rotation angle *θ*_KR_ can be understood with a model based on multiple Drude–Lorentz oscillators. For an exciton or trion resonance described by a Lorentz oscillator, the frequency dependence of *θ*_KR_ has the form


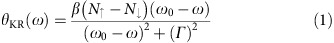


where *β* is a proportionality constant, *Γ* is the damping rate, (*N*_↑_−*N*_↓_) is the spin–valley polarization, that is, the population imbalance of excitons/trions in the K^+^ and K^−^ valleys, and *ω*_0_ the exciton/trion resonance frequency. The resulting curves for *θ*_KR_ exhibit a zero crossing at the exciton (trion) resonances. For GaAs-based heterostructures, it was shown that the sign of *β* is opposite for excitons and singlet trions^50^.

Owing to the fact that our WS_2_ monolayer is deposited on a silicon wafer with a top SiO_2_ layer, we need to consider multiple reflections at the interfaces and their effects on the Kerr spectrum, which have been shown to be substantial in various semiconductor heterostructures^51^,^52^. To this end, we measured the reflectance contrast of our sample structures using a white-light source (Supplementary Fig. 2 and Supplementary Note 2), and modelled it using three Lorentzians (corresponding to the neutral A and B excitons and the charged A exciton (trion)) in combination with the transfer matrix method, which accounts for multiple reflections. The same *Ansatz* is used to model the Kerr spectrum, for which we have to consider two trion species, however, and slightly shift the energetic positions of the resonances to account for variations between the samples used for the Kerr rotation and the reflectance contrast measurements (see Supplementary Figs 3 and 4 and Supplementary Note 3 for details).

In the energy range of the A exciton, we clearly observe a pronounced Kerr rotation angle after the arrival of the circularly polarized pump pulse. Its amplitude is strongly dependent on laser energy, and we can identify two energy regions where large Kerr signals are observed, at around 2,060 (corresponding to the PL emission of the two trion species) and 2,090 meV (corresponding to the neutral A exciton). The energy dependence of the Kerr signals is more easily seen in the lower panel of Fig. 2a, while the feature at 2,060 meV shows a broad maximum of Kerr rotation angle, the higher-energy feature has a more complex shape: here a large maximum of Kerr rotation angle is observed, followed by a zero crossing and a sign change of the rotation angle as the energy is increased.

The experimental data in the spectral range of the A exciton (open circles) are in excellent agreement with a fit using the sum of three Lorentzian resonance functions (red solid line), two trion resonances, which we attribute to the singlet and triplet states, and one exciton resonance. The lineshapes of the resonances are somewhat distorted compared with equation (1) by the effects of multiple reflections, which are taken into account by our model. Our fit results in *ω*_0_^X^=2,086 meV, *ω*_0_^T,t^=2,059 meV and *ω*_0_^T,s^=2,050 meV (blue dashed lines in the lower panel of Fig. 2a). *ω*_0_^T,s^ and *ω*_0_^T,t^ correspond to the singlet and triplet trions, respectively, and *ω*_0_^X^ to the neutral A exciton. As indicated by the individual resonance curves (black dashed lines) in the lower panel of Fig. 2a, the two trion resonances have opposite signs of *β*, resulting in the broad *θ*_KR_ maximum around 2,060 meV and an absence of the zero crossing that is observed for the higher-energy exciton feature due to partial cancellation. The singlet–triplet splitting of 9 meV extracted from the fit in Fig. 2a is in good agreement with the value extracted from the PL measurements discussed above. The fit also yields the damping rates *Γ*, which are similar for the two trion species (*Γ*^T,s^=*Γ*^T,t^=20 meV) and larger than for the neutral A exciton (*Γ*^X^=13.3 meV). We note that the energetic positions of the excitonic resonances do not shift as a function of the time delay Δ*t*, indicating that bandgap renormalization effects do not need to be considered for the excitation density used in our TRKR experiments (see Supplementary Fig. 5 and Supplementary Note 4 for details).

In the energy range of the B exciton, we observe a more simple dependence of *θ*_KR_ on laser energy, with a broad maximum centred at about 2,460 meV. As discussed above, we only need to utilize a single excitonic resonance in the energy range of the B exciton to accurately model the reflectance contrast of our sample. This is also the case for the Kerr rotation spectrum, as evidenced by the good agreement between the fit and the experimental data shown in the lower panel of Fig. 2b. Here the fit yields a B exciton resonance energy of 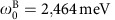 and a damping rate *Γ*^X^=41 meV. The spectral width of the Kerr resonance leads to a significant modification of the lineshape due to multiple reflections, so that only a single lobe remains, in stark contrast to the spectrally narrow A exciton Kerr resonance, which is only slightly distorted compared with the lineshape defined by equation (1). We note that here we neglect the possible influence of charged B excitons, which have been reported recently^53^, on the Kerr rotation spectrum, as we can expect their contribution to the signal to be smaller than that of the neutral exciton, similar to the A exciton energy range, due to the small carrier concentration in our sample. Given the spectral width of the B exciton resonance, which extends over the exciton–trion splitting, the B trion resonance would be very hard to discern.

### A exciton valley polarization dynamics

**Figure 3:**
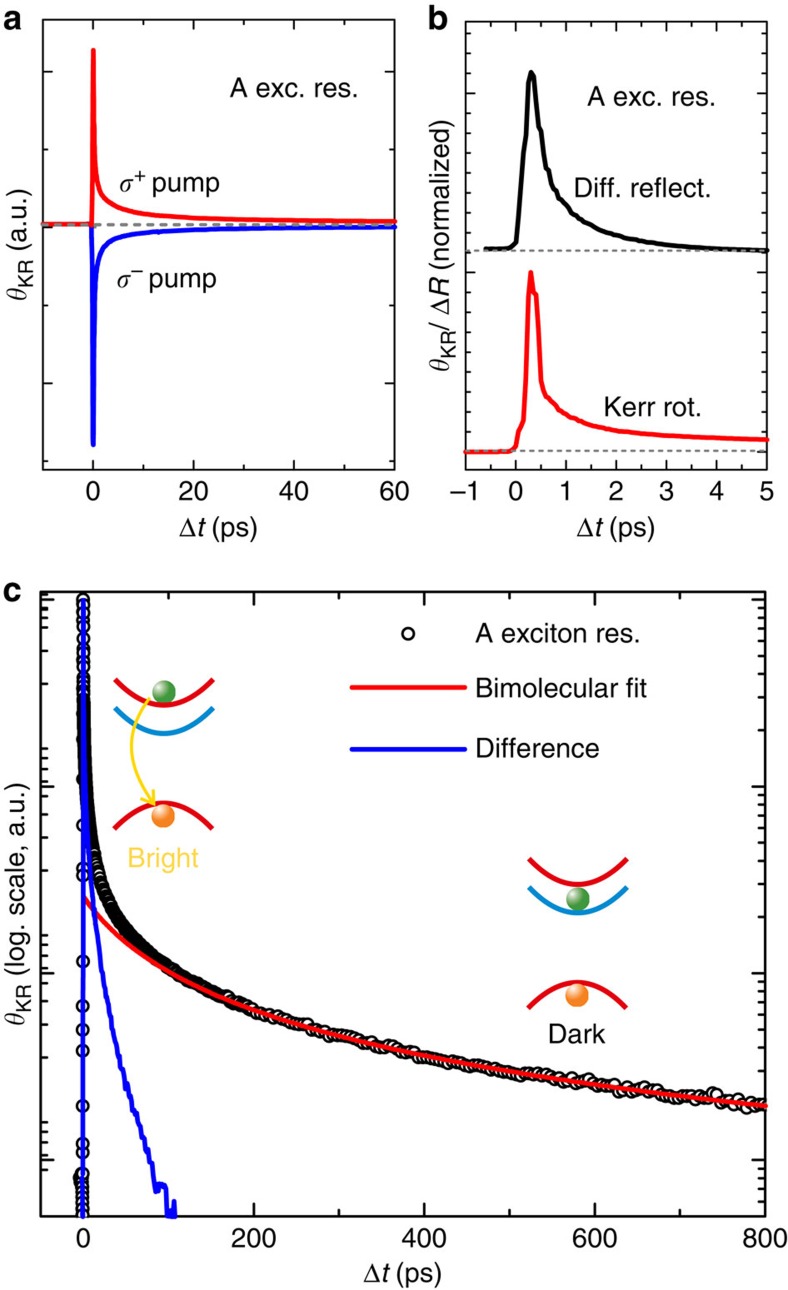
A exciton valley polarization dynamics. (**a**) Helicity dependence of TRKR signal for the laser energy tuned to the A exciton resonance (exc. res.). (**b**) Direct comparison of differential reflectivity (Diff. reflect.) and TRKR traces (Kerr rot.) on the few-picosecond timescale at the A exciton resonance energy. (**c**) Log. scale plot of TRKR traces at the A exciton resonance (A exciton res.) on the 100 ps timescale. A bimolecular fit to the data (open circles) is indicated by the solid red line. The difference between this fit and the data is indicated by the solid blue line. The insets sketch the configuration of optically bright (dark) excitons, which dominate the Kerr signal at early (late) delay times Δ*t*.

We now turn to the dynamics of the TRKR traces. As Fig. 3a shows, the TRKR signal depends on the helicity of the pump beam, and its sign flips as the helicity of the pump beam is changed from *σ*^+^ to *σ*^−^. For all other TRKR measurements shown here, two traces with opposite pump helicities are recorded in sequence, and the difference of these traces is calculated. This scheme ensures that any remaining time-resolved signal that is not helicity-dependent cancels out. To understand the dynamics of the TRKR traces, we need to consider the different quantities measured in (time- and) helicity-resolved PL measurements and TRKR traces. In helicity-resolved PL, the circular polarization degree of the emitted PL is detected, which is directly related to the valley polarization degree in monolayer WS_2_ via the optical selection rules. In the absence of processes which lead to spin–valley relaxation, the circular polarization degree of the PL will remain constant throughout the exciton lifetime. By contrast, Kerr rotation measures the spin–valley polarization, that is, the density of polarized excitons or trions within the probe spot area. Hence, a reduction of the exciton density via photocarrier recombination will lead to a decay of the Kerr signal, even in the absence of any spin–valley relaxation processes.

Given that the radiative recombination in monolayer dichalcogenides has been shown to be very fast^8^,^54^, this process cannot be neglected in the interpretation of the Kerr signals. Therefore, we also utilize time-resolved differential reflection measurements to study the exciton population dynamics at short timescales. In Fig. 3b, we directly compare Kerr rotation and differential reflectivity traces for the A exciton resonance on the few-picosecond timescale. We clearly observe a very rapid partial decay of the signal within about 200 fs in, both, the Kerr rotation and the differential reflectivity trace. This value is in good agreement with a previous study on the radiative lifetime in monolayer WSe_2_ (ref. 8), which was found to be about 150 fs.

This rapid radiative recombination is only possible for excitons with a sufficiently small centre-of-mass momentum **k** to match the light cone. While we resonantly excite excitons at **k**=0, exciton–exciton and exciton–phonon scattering processes rapidly lead to a redistribution in phase space and a population of higher-**k** states.

After the initial rapid decay, the differential reflectivity and Kerr rotation traces show markedly different dynamics: the differential reflectivity trace can be described with a biexponential fit consisting of the rapid, 200 fs component and a slower component with a decay constant of a picosecond that we can relate to phase space distribution via scattering processes. By contrast, the Kerr rotation trace has a more complex time dependence that can only be described by a triple exponential fit on the few-picosecond timescale. For the A excitons in WS_2_, we need to consider multiple processes that lead to this complex time dependence: due to the background doping in our sample, a resonantly excited exciton can capture an additional electron to form a trion. As can be seen from the fit functions in the lower panel of Fig. 2a, both the singlet and the triplet trion states will yield a finite Kerr angle when probed at the exciton energy, with smaller amplitude and opposite sign. Thus, trion formation will lead to a reduction of the Kerr signal, as the Kerr response shifts from resonant probing of excitons to off-resonant probing of trions. Very recently, it was shown that trion formation after resonant excitation of excitons occurs on the 2 ps timescale in monolayer MoSe_2_ (ref. 55), we can therefore expect a similar value for WS_2_, making trion formation a relevant process for the decay of the Kerr signal. In addition, we need to consider the peculiar spin splitting of the WS_2_ conduction band: here the upper spin-split conduction band is addressed in the A exciton transition, and either intravalley spin-flip transitions or (spin-conserving) intervalley transitions^22^ from the upper to the lower conduction-band state are energetically favourable, yielding another decay channel. Experimental evidence for the relevance of these processes was recently found in temperature-dependent PL measurements on WSe_2_, for which calculations^21^ yield a conduction-band splitting similar to WS_2_: for the neutral exciton, the PL yield increased with temperature, indicating a rapid relaxation from the upper to the lower conduction-band state after nonresonant excitation, and a thermally activated transfer into the upper conduction-band state^23^,^26^.

A conduction-band spin flip will switch the A exciton to an optically dark state, blocking radiative recombination. For this optically dark state, radiative recombination via the intermediate state of capturing an additional electron to form an optically bright trion is also suppressed, as the resident electrons occupy the lower spin-split conduction-band states at low temperatures, so that only an optically dark intervalley singlet trion may form. Further evidence for the formation of these dark exciton states may be found by studying the Kerr traces at larger time delays. Owing to the high signal-to-noise ratio of our Kerr rotation experiment, we are able to study the evolution of the Kerr trace at the exciton resonance over more than two orders of magnitude. Figure 3c shows this Kerr trace (open circles) on a logarithmic scale for a large time range of 800 ps. While the signal initially decays very rapidly due to the mechanisms discussed above, we find that for time delays larger than 150 ps, the remaining signal decay is very well described by a bimolecular fit function (red solid line), which indicates a decay mediated by exciton–exciton interaction^8^. The difference (solid blue line) between this bimolecular decay and the experimental data becomes negligible, indicating that all of the fast processes discussed above are inactivated. This finding indicates that during the first 150 ps, a subset of optically dark excitons is created, which still gives rise to a finite Kerr signal due to the presence of the valley-polarized holes in the upper valence band. The decay of this subset occurs via exciton–exciton interaction-driven Auger recombination, which was previously observed to be efficient in WSe_2_ monolayers^8^.

### Valley dynamics at the A and B exciton resonances

**Figure 4:**
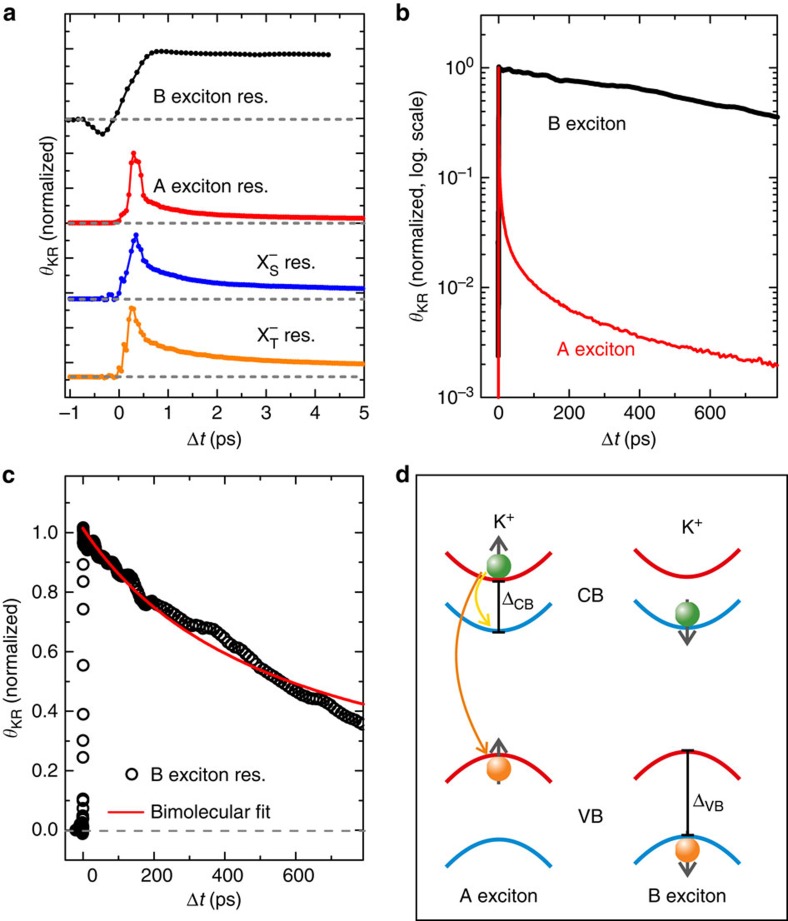
Valley polarization dynamics of A and B excitons. (**a**) Direct comparison of TRKR traces on the few-picosecond timescale at A exciton resonance (A exciton res.), trion resonance (X^−^_S_res. and X^−^_T_res.) and B exciton resonance (B exciton res.) energies. (**b**) Comparison of A and B exciton TRKR traces on the 100 ps timescale with logarithmic amplitude scale. (**c**) TRKR trace at the B exciton resonance. A bimolecular fit to the data (open circles) is indicated by the solid red line. (**d**) Schematic illustration of A and B exciton configurations for WS_2_ in the K^+^ valley. The yellow and orange arrows indicate the conduction-band splin-flip and radiative recombination processes leading to the decay of the A exciton Kerr signal.

Finally, we discuss the dynamics of TRKR traces measured for different excitation energies. Figure 4a shows a direct comparison of the TRKR traces measured at the A exciton, singlet/triplet trion and B exciton resonance energies on the few-picosecond timescale. To improve the signal-to-noise ratio of the B exciton trace, we averaged several traces measured at different energies around the maximum Kerr angle (Fig. 2b). To separately probe either the singlet or the triplet trions, we tune our laser system to the energies where the Kerr amplitude of the other trion state has its zero crossing according to our fit to the Kerr spectra (Fig. 2a). Direct comparison of the A exciton and trion traces indicates a larger long-lived signal component for the two trion states. This is expected, as the process of trion formation discussed above, which contributes to the rapid decay of the neutral exciton signal, is obviously absent for trion-resonant excitation and probing. By contrast, either intravalley spin-flip transitions (for the triplet trion) or spin-conserving intervalley transitions (for the singlet trion) from the upper to the lower conduction-band state are energetically favourable. In either case, the resulting state is an optically dark intervalley singlet trion. While we need to consider some admixture of singlet and triplet Kerr signals due to the finite spectral linewidth of the laser system, we find a larger long-lived component of the Kerr trace for the triplet, in qualitative agreement with the larger PL circular polarization degree of the triplet emission discussed above (Fig. 1b). This finding also suggests that the conduction-band intervalley scattering process discussed for the singlet trion has a higher rate than the intravalley spin-flip transition for the triplet trion. More remarkably, there is a striking difference in the dynamics of A and B exciton TRKR traces: on the few-picosecond timescale, the B exciton trace does not show complex, rapid decay dynamics, but a simple step-like behaviour. This indicates that the decay processes discussed for the A exciton, sketched in Fig. 4d, are not active for the B exciton: naturally, since the B exciton transition addresses the lower spin-split conduction band, conduction-band spin-flip processes are not energetically favourable. Remarkably, in contrast to the A exciton, there is also no contribution of rapid photocarrier recombination to the Kerr signal decay. This observation is corroborated by the absence of pronounced B exciton PL emission in low-temperature PL measurements, even under pulsed excitation (Supplementary Fig. 6 and Supplementary Note 5).

The different dynamics of TRKR traces at A and B exciton resonance becomes even more evident when we investigate the behaviour on longer timescales, as shown in Fig. 4b. While the A exciton trace decays by more than two orders of magnitude in the first 100 ps, due to radiative recombination, trion formation and conduction-band spin-flip processes, the B exciton Kerr signal only decreases by about 15% in the same time interval. In contrast to the A exciton, the B exciton trace also does not show a crossover of the decay dynamics from rapid to slow processes, indicating that a single mechanism governs the Kerr signal decay of the B exciton.

As depicted in Fig. 4c, which shows the B exciton Kerr trace dynamics on a linear scale, a bimolecular decay function yields reasonable agreement to the experimental data, indicating that the decay may be driven by exciton–exciton annihilation/Auger recombination of B excitons.

We note that there are alternative interpretations for the dynamics shown in Fig. 4c: one possibility would be the relaxation of the hole bound in the B exciton from the lower to the upper valence band, either in a spin-conserving intervalley transition, or in an intravalley spin-flip transition. This would transform a B exciton into an optically dark A exciton state, since the electron is excited into the lower conduction band via the B exciton transition. This valence-band relaxation would therefore lead to the bimolecular decay dynamics discussed above for the A exciton transition.

Another alternative would be the transfer of spin polarization to resident electrons. In our sample, the background carrier concentration, extracted from the exciton–trion splitting (Supplementary Note 1), is so low that only the lower conduction band is occupied at low temperatures. Since the B exciton transition directly addresses the lower conduction band, an effective transfer of spin polarization from B excitons to resident electrons would be possible if the valley polarization of holes in the lower valence band is lost during energy relaxation into the upper valence band. These unpolarized holes could eventually recombine with electrons from both valleys, yielding an excess of spin- and valley-polarized resident electrons. Analogous processes for transferring spin polarization from optically oriented electron–hole pairs to resident carriers have been observed in GaAs-based heterostructures^47^, and their efficiency hinges on an imbalance of the spin dephasing times for electrons and holes. Given that spin lifetimes of several nanoseconds have recently been reported for resident electrons even in highly doped WS_2_ monolayers^44^,^45^, the slow decay of the Kerr signal at the B exciton resonance might thus be attributed to a transfer of spin polarization to resident carriers.

For either of the two alternative interpretations discussed above, the Kerr signal would arise from the valley occupation imbalance of electrons in the lower conduction band probed via the B exciton transition. However, either of these alternative processes would also lead to a partial decay of the Kerr signal due to the relaxation of valley-polarized holes, similar to the effects of conduction-band transitions discussed for the bright A exciton. These processes are expected to lead to non-trivial dynamics directly following the excitation and would thus require a more detailed study at ultra-short timescales, which are below the temporal resolution in our experiment.

The long-lived Kerr rotation signal we observe when resonantly exciting the B exciton is highly interesting for potential valleytronic applications. In stark contrast to the A exciton and trion states, however, a direct read-out of the B exciton valley polarization via PL measurements is not possible.

In conclusion, we have investigated monolayer WS_2_ samples by means of PL spectroscopy, as well as TRKR measurements at low temperatures. In PL measurements, we are able to resolve two different optically bright trion states, which we interpret as intravalley singlet and intervalley triplet trions, with an energy splitting of about 10 meV due to exchange interaction. These states also differ in their steady-state circular polarization degree, indicating different valley dephasing rates. Clear evidence for these different trion states is also obtained in energy-dependent TRKR measurements. Furthermore, we utilize TRKR to extract the coupled spin–valley dynamics for the neutral A and B excitons as well as the trion states. We observe a rapid initial decay of the valley polarization for the A exciton and the trions, and identify a peculiar crossover towards exciton–exciton interaction-related decay dynamics, consistent with the formation of a subset of optically dark A excitons. Remarkably, these dark excitons are directly observable in our TRKR measurements. By contrast, resonant excitation of the B exciton transition leads to a very slow decay of the Kerr signal, making this transition potentially interesting for future valleytronic applications. The difference between the A and B exciton spin–valley dynamics is directly related to the negative conduction-band spin splitting in the tungsten-based TMDC monolayers.

## Methods

### Sample preparation

WS_2_ flakes are prepared from mineral bulk crystals (HQgraphene) using a recently developed all-dry transfer technique^56^. The flakes are *n*-doped with a background carrier density of *n*_e_=1.7 × 10^12^ cm^−2^, which can be extracted from the exciton–trion splitting observed in optical spectroscopy (see Supplementary Note 1 for details). The size of the monolayer flakes ranges between 3,000 and 4,000 μm^2^, allowing for optical measurements with a large focal spot. We utilize a *p*-doped silicon wafer with 285 nm SiO_2_ layer and lithographically defined metal markers as final substrate. Supplementary Fig. 7 depicts micrographs of the investigated samples.

### Pump–probe spectroscopy

The TRKR and differential reflectivity (Δ*R*) measurements are performed with a tunable, frequency-doubled pulsed fibre laser system (Toptica TVIS, pulse length (full-width at half-maximum) 180 fs, spectral linewidth (full-width at half-maximum) 7 meV). The laser beam is split into pump and probe pulse trains, and a variable delay between the pulses is realized using a mechanical delay stage. Pump and probe beams are focussed onto the sample, which is mounted in vacuum on the cold finger of a He-flow cryostat, to a spot size of about 30 μm, using an achromatic lens. Excitation densities of about 50 and 200 W cm^−2^ are used in the pump and probe beams, respectively. For an excitation energy resonant with the A exciton transition, this yields a maximum exciton density of about 2.5 × 10^11^ cm^−2^.

A digital microscope system allows to bring the laser focus onto the TMDC flakes. For TRKR measurements, the pump beam is circularly polarized using an achromatic quarter-wave plate, while the probe beam is linearly polarized. To detect changes of the reflected probe beam polarization state, an optical bridge detector is used. For all TRKR measurements, two traces with opposite pump helicities are recorded in sequence, and the difference of these traces is calculated. This scheme ensures that any time-resolved signal that is not helicity-dependent cancels out. Δ*R* measurements are performed in the same manner, however, the pump and probe beams are linearly polarized orthogonal to each other, and the intensity of the reflected probe beam is detected using a photodiode. All TRKR and differential reflectivity measurements described in this manuscript are performed at a nominal sample temperature of 4.5 K.

### PL spectroscopy

For the PL measurements, we use a frequency-doubled continuous-wave solid-state laser emitting at 561 nm. The light is focussed on the sample via a microscope objective to a spot size of ∼4 μm, and the backscattered PL is analysed in a spectrometer with a 1,200 lines per mm grating and a liquid-nitrogen-cooled charge-coupled device chip. For these measurements, the sample is mounted in a cryostat in which He exchange gas is used for cooling. All PL measurements described in this manuscript are performed at a nominal sample temperature of 4.5 K.

### Data availability

The data that support the findings of this study are available from the corresponding author on request.

## Additional information

**How to cite this article:** Plechinger, G. *et al.* Trion fine structure and coupled spin–valley dynamics in monolayer tungsten disulfide. *Nat. Commun.* 7:12715 doi: 10.1038/ncomms12715 (2016).

## Supplementary Material

Supplementary Information Supplementary Figures 1-7, Supplementary Notes 1-5, Supplementary Methods and Supplementary References.
