# Supplementary material for: Trion fine structure and coupled spin–valley dynamics in monolayer tungsten disulfide
Source: Nat Commun. 2016 Sep 2;7:12715. doi: 10.1038/ncomms12715 (PMC5025800; doi:10.1038/ncomms12715)
Supplement: Supplementary Information — Supplementary Figures 1-7, Supplementary Notes 1-5, Supplementary Methods and Supplementary References. [file ncomms12715-s1.pdf]

Trion fine structure and coupled spin-valley dynamics in monolayer Tungsten Disulfide - Supplementary Information -

I. SUPPLEMENTARY FIGURES

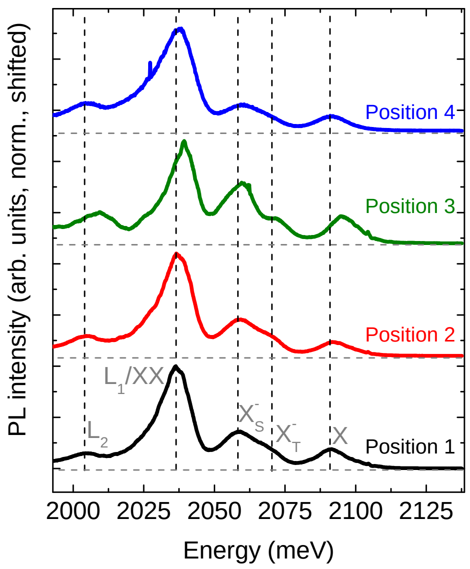

Supplementary Figure 1. **Position-dependence of trion fine structure in Photoluminescence.** Comparison of PL spectra recorded on different positions of a monolayer WS_2 flake. All spectra were measured at 4 K. Position 1 corresponds to the position for which the spectrum is shown in Figure 1a of the main manuscript. Vertical dashed lines serve as guide to the eye. The trion fine structure is visible on all 4 positions.

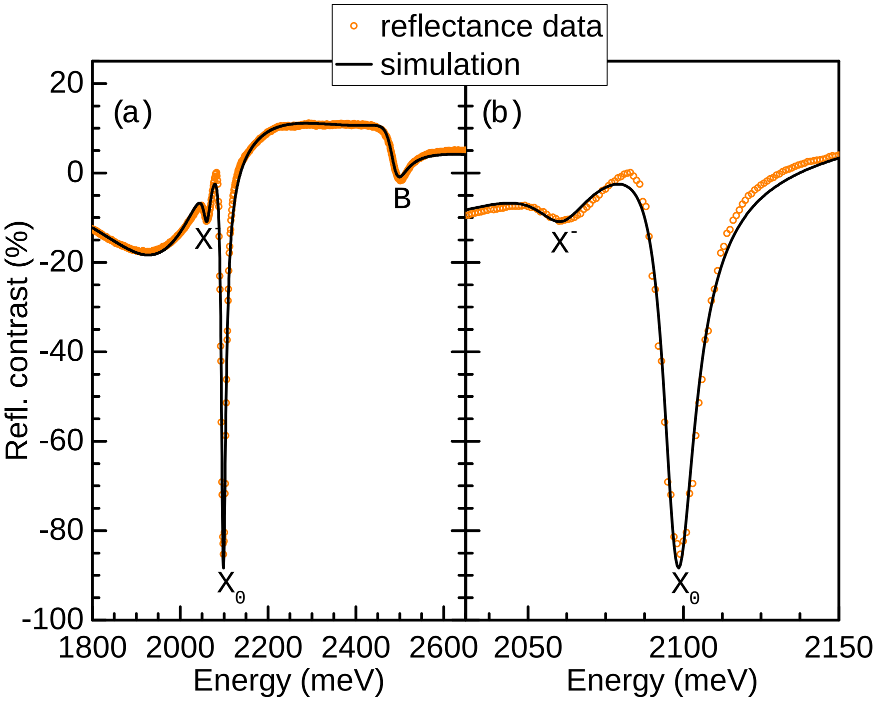

Supplementary Figure 2. **Low-temperature reflectance spectra.** White-light reflectance spectrum of a monolayer WS_2 flake measured at 4 K (orange circles) and fit to the data (black solid line). X and B mark the excitonic resonances corresponding to the A and B excitons, X^- marks the trion resonance. Panel (a) shows a large spectral range containing, both, A and B exciton resonances, while panel (b) shows a smaller spectral range of the same dataset in the vicinity of the A exciton resonance.

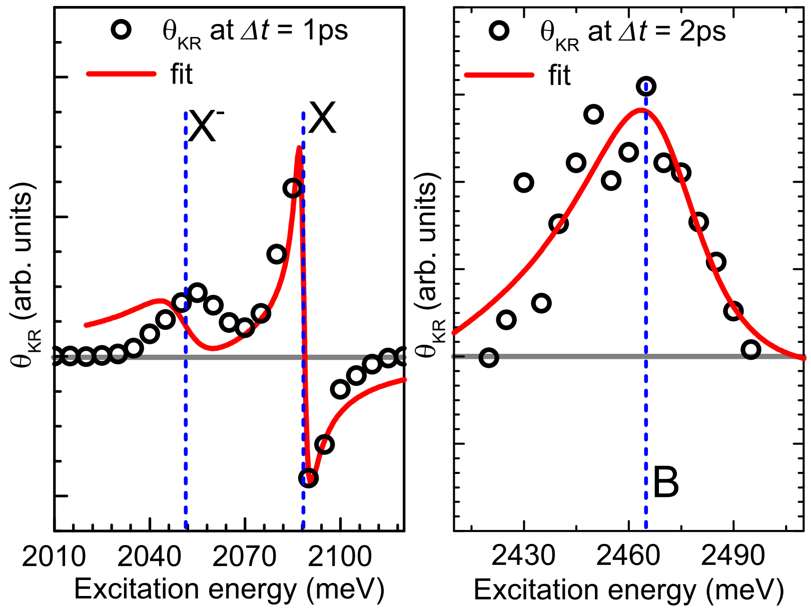

Supplementary Figure 3. **Modeling of experimental Kerr spectra.** Experimental Kerr rotation data (circles) and fit (red solid line) based on one trion and two exciton resonances shown in the range of the A exciton (left panel) and the B exciton (right panel).

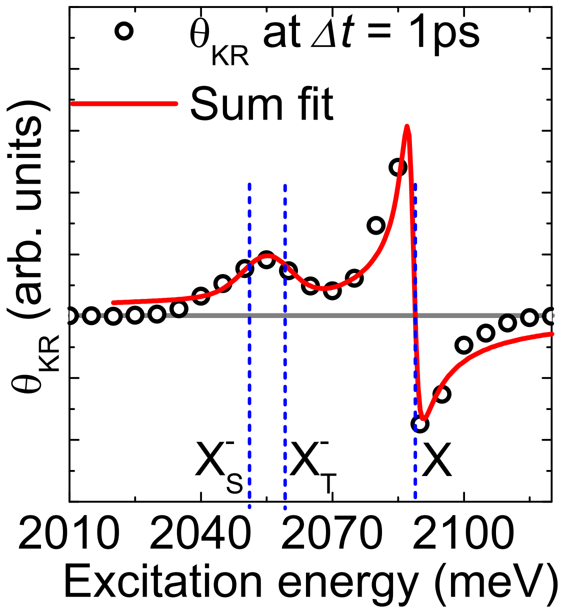

Supplementary Figure 4. **Improved fit of trion resonances in Kerr spectra.** Experimental Kerr rotation data (circles) and improved fit (red solid line) utilizing two trion resonances shown in the energy range of the A exciton.

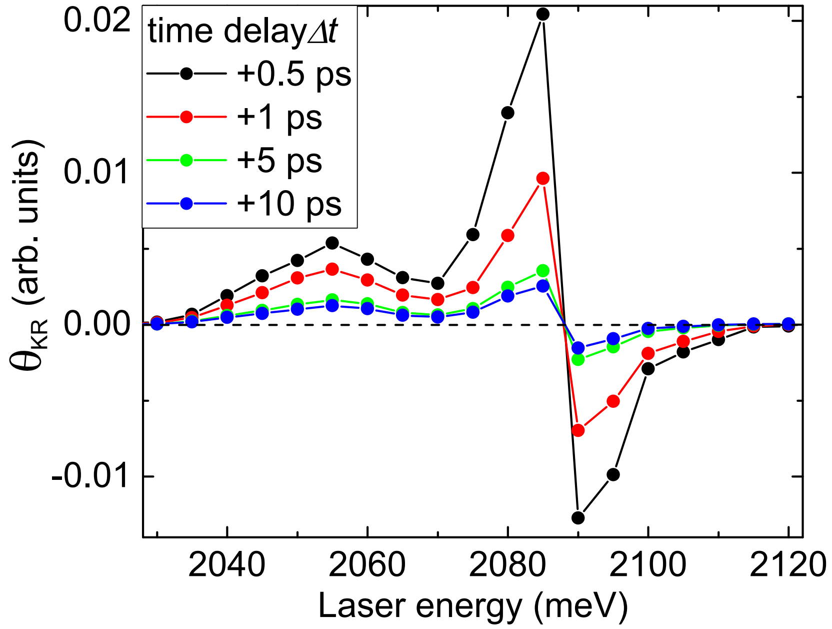

Supplementary Figure 5. **Temporal evolution of Kerr spectra.** Kerr rotation spectra extracted from the dataset in Figure 2a of the main manuscript.

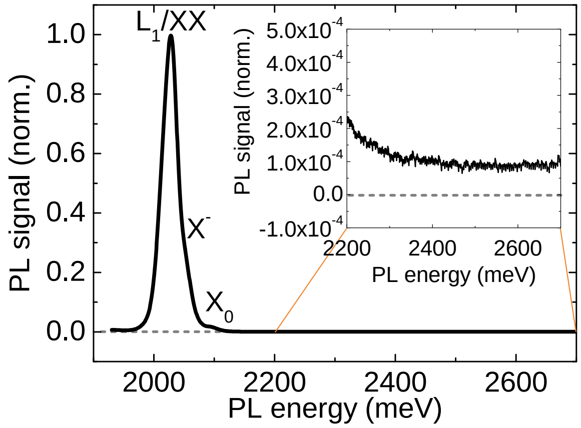

Supplementary Figure 6. **Photoluminescence under UV excitation.** PL spectrum of monolayer WS_2 flake measured at 4 K using pulsed UV laser excitation. The inset shows the spectral region where the B exciton resonance is observed in Kerr spectroscopy.

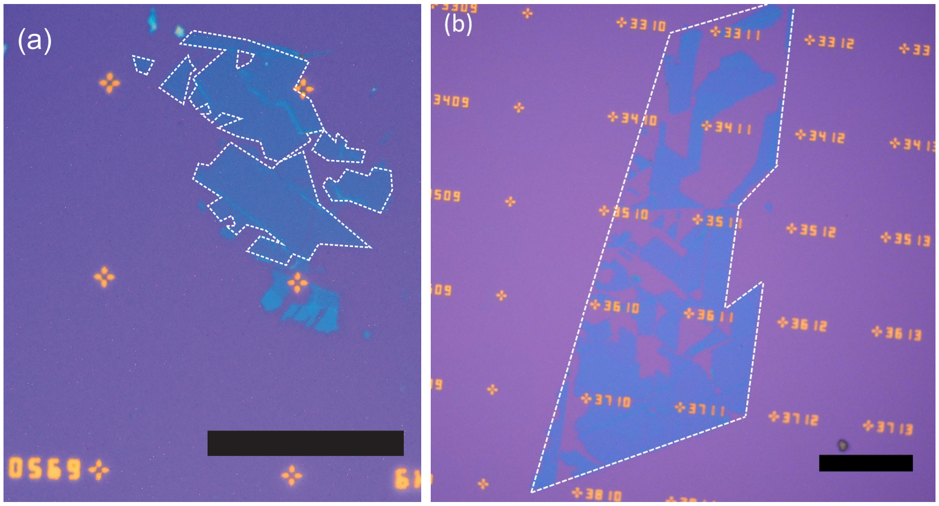

Supplementary Figure 7. **Sample images.** (a) Optical micrograph of the WS_2-flake used for PL measurements. (b) Optical micrograph of the WS_2-flake used for TRKR measurements. The main part of the area marked by the white dashed line is covered by monolayer material originating from a big single-crystalline flake. The scale bar is 50 µm.

II. SUPPLEMENTARY NOTES

A. Supplementary Note 1: Calculation of background carrier concentration in our samples

To extract the background carrier density, we utilize the fact that in the TMD monolayers, the exciton-trion splitting ΔXT observed in optical spectroscopy sensitively depends on the chemical potential (Fermi energy E_{F}) [1]. This is due to the fact that for resonant creation (probed in absorption-related experiments like Kerr spectroscopy) or annihilation (probed in PL experiments) of a trion, energy and momentum conservation have to be taken into account [2]. We can extract this splitting directly from our PL and energy-dependent Kerr spectra. Here, in order to be able to compare our results directly to literature data, we neglect the fact that we are able to observe a fine structure splitting of the trion in our measurements, and extract the energetic position of the trion by (a) using a single Gaussian to fit its spectral position in the PL spectrum and (b) calculating the weighted average of the energetic positions of the two trion resonances in the fit to the energy-dependent Kerr spectrum. We find an exciton-trion separation of 31 meV (PL data) and 31.5 meV (Kerr data). Following the reasoning presented by Chernikov et al. [2], we can calculate the Fermi energy according to

$$\Delta XT = \Delta_0 + \frac{2}{3} E_{\mathrm{F}} \tag{1}$$

Here, Δ_0 is the trion binding energy in the limit of zero background carrier density. Using the value of $\Delta_0 = 23 \pm 1$ meV [2], we find that $E_{\mathrm{F}} = 12 \pm 3$ meV. For a two-dimensional electron gas, carrier density and Fermi energy are related by

$$n_{\mathrm{e}} = \frac{g_{\mathrm{V}} m^*}{2\pi\hbar^2} E_{\mathrm{F}} \tag{2}$$

Here, $g_{\mathrm{V}} = 2$ is the valley degeneracy factor. Using a conduction-band mass of $m^* = 0.3 \times m_{\mathrm{e}}$, it follows that $n_{\mathrm{e}} = (1.7 \pm 0.4) \times 10^{12}$ cm^{-2}. The carrier density for which the spin-split upper conduction band becomes occupied has been calculated by Kormányos et al. [3] to be about 4.7×10^{12} cm^{-2}, well above the value of background carrier density extract from our calculations above.

B. Supplementary Note 2: Low-temperature white-light reflectance spectrum of monolayer WS_2

Supplementary Figure 2 shows a white-light reflectance contrast measurement recorded on a monolayer WS_2 flake on top of a silicon substrate with a 285 nm thick SiO_2 oxide layer. The spectrum was measured at 4 K (see Supplementary Methods for details of the white-light reflectance experiment). Subsequent measurements of the energy-dependent intensity of the light reflected by the bare silicon/SiO_2 substrate $I_{\mathrm{Sub}}(E)$ and by the monolayer WS_2 flake $I_{\mathrm{WS}}(E)$ on top of the substrate were recorded, and the reflectance contrast spectrum $R(E)$ depicted in Supplementary Figure 2 was calculated from these measurements via:

$$R(E) = \frac{I_{\mathrm{WS}}(E) - I_{\mathrm{Sub}}(E)}{I_{\mathrm{WS}}(E) + I_{\mathrm{Sub}}(E)}. \tag{3}$$

In order to describe the reflectance spectrum, we need to utilize a transfer matrix method approach [4] to account for multiple reflections at the interfaces of our SiO_2/Si substrate. By assuming three excitonic resonances, we are able to get excellent agreement between our data and our model. We find the following energetic positions for the excitonic resonances: neutral A exciton X^0: 2096 meV (FWHM: 13.3 meV), charged exciton X^-: 2060 meV (FWHM: 21.5 meV), neutral B exciton: 2484 meV (FWHM: 39.3 meV). We note that the charged exciton X^- resonance FWHM significantly exceeds the trion fine structure splitting observed in the main manuscript, so that this effect is not discernible in the reflectance measurement. The exciton-trion splitting in this sample corresponds to a Fermi energy of 19.5 ± 3 meV, which yields a carrier density of $n_{\mathrm{e}} = (2.7 \pm 0.4) \times 10^{12}$ cm^{-2}.

C. Supplementary Note 3: Substrate-induced effects on energy-dependent Kerr rotation spectrum

As shown in supplementary Note 2, we are able to account for the effects of multiple reflections due to the silicon/SiO_2 substrate by means of a transfer matrix approach. We apply this approach to model the energy dependence of the Kerr rotation angle as well, following the procedure described here [5]. For this, we need to introduce another variable in addition to the oscillator strength of the individual resonances, namely the valley polarization of each resonance, which is created by the circularly polarized pump in the experiment. Additionally, we need to consider that the spectral positions of the individual resonances

extracted from the white-light reflectance measurement may need to be shifted slightly to account for small differences between the flake used in that measurement and in the Kerr rotation measurements. Supplementary Figure 3 shows the results of a fit to the Kerr rotation data which takes these considerations into account, using three resonances, a trion, a neutral A exciton and a neutral B exciton. To match the resonances observed in the Kerr rotation data, we red-shifted the spectral positions for the A exciton and its trion by 10 meV, and that of the B exciton by 20 meV. As the figure shows, this fit is in good agreement with the experimental data in the range of the neutral A and B excitons. However, it fails to describe the feature we associate with the trions. To improve our fit, we utilize two trion resonances corresponding to the two trion species observed in our PL experiments. For this, we assume that the antisymmetric Lorentzians for the two trion species have opposite signs of β, but comparable damping rates $\mathit{\Gamma}$. As shown in Supplementary Figure 4, this fit captures all the salient features of the experimental data. It also allows us to extract the energetic splitting between the two trion species, which we find to be in good agreement with the value found in our PL experiments.

D. Supplementary Note 4: Time-dependence of exciton Kerr resonance

To investigate possible bandgap renormalization effects as a function of exciton density, we look more closely at our energy-dependent Kerr rotation spectra. Supplementary Figure 5 compares energy-dependent Kerr rotation data for different time delays, extracted from the dataset shown in Figure 2(a) of the main manuscript. We clearly see that the spectral positions of the resonances do not change as function of delay time. We note that in the time interval from $\mathit{\Delta} t = 0.5$ ps to $\mathit{\Delta} t = 10$ ps, the Kerr signal decays by almost one order of magnitude, and a substantial part of this decay is due to photocarrier recombination, so that the exciton density is also significantly reduced from its initial value. If there were exciton-density-dependent bandgap renormalization effects in the density range that we create, we would expect to see a time-dependent shift of the Kerr resonances.

E. Supplementary Note 5: Low-temperature photoluminescence of monolayer WS_2 under pulsed UV excitation

Supplementary Figure 6 shows a micro-PL spectrum of a monolayer WS_2 flake measured at 4 K using pulsed UV laser (wavelength 355 nm, pulse length about 50 ps, repetition rate 80 MHz) excitation (see Supplementary Methods for further details of the PL experiment). The spectrum is dominated by the biexciton feature marked XX, as expected for high carrier densities, the charged exciton states are visible as a high-energy shoulder of the XX state, and a relatively weak neutral A exciton peak is also observed. In the spectral range where the B exciton resonance is observed in our Kerr spectroscopy and white-light reflectance experiments, no PL emission is observable, as the inset shows.

III. SUPPLEMENTARY METHODS

A. Photoluminescence

For the PL measurements shown in Supplementary Figure 6, we utilize a picosecond pulsed diode laser system (Picoquant LDH-P-FA-355) emitting at 355 nm in conjunction with a self-built confocal microscope system. The laser was coupled into a 100x objective and focussed to a spot size of about 1 μm on the sample surface. The PL was collected by the same objective and recorded using a single-grating spectrometer equipped with a CCD sensor. A low-pass filter with an onset at a wavelength of 400 nm was used in front of the spectrometer slit to suppress stray light from the laser. The sample was mounted in a small He-flow cryostat.

B. White-light reflectance

For white-light reflectance measurements, the output of an incandescent lamp was focussed onto a pinhole, and an achromatic lens was used to collimate the emission from the pinhole. This was coupled into a self-built microscope setup with a 100x objective lens, so that the white light could be focussed down to a spot size of about 4 μm on the sample surface. The sample was mounted in a small He-flow cryostat. The reflected light was collected with the same objective lens and coupled into a spectrometer, where it was detected with a

Peltier-cooled CCD.

SUPPLEMENTARY REFERENCES

[1] K. F. Mak et al., "Tightly bound trions in monolayer MoS_2," Nature Materials **12**, 207–211 (2013).

[2] A. Chernikov et al., "Electrical tuning of exciton binding energies in monolayer WS_2," Physical Review Letters **115**, 126802 (2015).

[3] A. Kormányos et al., "$k \cdot p$ Theory for Two-Dimensional Transition Metal Dichalcogenide Semiconductors," 2D Materials **2**, 022001 (2015).

[4] E. Hecht, *Optics* (Addison-Wesley, Reading, 1998).

[5] A. Arora, A. Mandal, S. Chakrabarti, and S. Ghosh, "Magneto-optical kerr effect spectroscopy based study of Landé g-factor for holes in GaAs/AlGaAs single quantum wells under low magnetic fields," Journal of Applied Physics **113**, 213505 (2013).
